# Transvaginal Retropubic Versus Transobturator Midurethral Sling in the Treatment of Recurrent Stress Urinary Incontinence

**DOI:** 10.7759/cureus.76480

**Published:** 2024-12-27

**Authors:** Lisandra Mendonça, Dora Antunes, Inês Coutinho, Liana Negrão, Fernanda Águas

**Affiliations:** 1 Gynecology/Obstetrics, Unidade Local de Saúde de Viseu Dão-Lafões, Viseu, PRT; 2 Gynecology and Obstetrics, Unidade Local de Saúde de Coimbra, Coimbra, PRT; 3 Gynecology and Obstetrics, Faculty of Medicine, University of Coimbra, Coimbra, PRT; 4 Gynecology, Unidade Local de Saúde de Coimbra, Coimbra, PRT

**Keywords:** efficacy, midurethral sling, patient outcome assessment, postoperative complications, urinary incontinence

## Abstract

Introduction: Transvaginal retropubic (TVT-R) and transobturator (TVT-O) midurethral slings are the main surgical options for stress urinary incontinence (SUI). Surgical indications for each of them are defined by clinical and history presentation. These techniques play a particular role in SUI recurrence after a previous urinary incontinence surgery, although there are few studies comparing their efficacy. This study aims to compare the outcomes of TVT-R versus TVT-O sling procedures in patients with recurrent/persistent SUI who were submitted to a previous incontinence surgery.

Methods: Retrospective and comparative study including all patients submitted to a repeated midurethral sling procedure due to recurrent/persistent SUI between January 1st 2019 and December 31st 2023 at the Gynecology Department of Unidade Local de Saúde (ULS) of Coimbra, Portugal. Demographic and clinical characteristics, surgical efficacy, and intra and postoperative complications were collected through medical records. At least eight months of follow-up were accomplished. Statistical analysis was performed using SPSS software, version 26.0 (IBM Corp., Armonk, NY), considering a significant p-value <0.05.

Results: Overall, 860 women were submitted to a midurethral sling procedure, of which 806 (93.7%) placed a transobturator sling and 54 (6.3%) a retropubic sling. Of these, 42 underwent repeated incontinence surgery due to recurrent/persistent SUI, of which 28 (66.7%) TVT-R and 14 (33.3%) TVT-O. No statistically significant differences were found between patients undergoing TVT-R vs TVT-O, considering the median age, body mass index, parity, and postmenopausal status (p=non significant (n.s.)*)*. There was a significant difference in urethral mobility prior to surgery between groups, with most TVT-R patients having fixed urethra and most TVT-O patients having mobile urethra (p<0.001)*. *The rate of intraoperative and immediate postoperative complications was similar in both groups (10.7% vs 7.1%, p=n.s.*)*. Bladder laceration was the most common complication, reported only in the TVT-R group. There was a complete resolution of SUI complaints after surgery in 75.0% and 85.7% of cases, respectively (p=n.s.*). *Long-term complications were also similar in both groups (21.4% vs 14.3%, p=n.s.*)*, with a worsening/appearance of urge urinary incontinence in 17.9% *vs *21.4% of cases (p=n.s.).

Conclusion: In the present study, retropubic midurethral sling was the most performed procedure in the treatment of SUI recurrence after previous incontinence surgery. Efficacy rates in SUI treatment were high in both groups, with no statistically significant differences between the two surgical techniques. Although it was expected that TVT-R would result in a higher rate of complications given its greater surgical complexity, there were no differences between both groups in terms of intra and postoperative complications. Therefore, we should select the surgical option that provides the best conditions for the treatment of recurrent/persistent SUI, avoiding major complications and in accordance with the clinical assessment and the patient’s preference.

## Introduction

Stress urinary incontinence (SUI) is defined by the International Continence Society (ICS) as “the complaint of any involuntary loss of urine on effort, physical strain, or when coughing or sneezing” [1]. Although the exact etiology of SUI is unclear, common explanations include the absence of urethra coaptation, muscular compression of the proximal urethra, and stabilization of the bladder neck and proximal urethra that allows equal pressure transmission of increased abdominal pressure [2].

Midurethral synthetic sling (MUS) procedures, including both retropubic tension-free vaginal tape (TVT-R) and transobturator vaginal tape (tension-free vaginal tape obturator (TVT-O) or transobturator tape (TOT) are considered the gold-standard surgical treatments for moderate to severe female SUI with reasonable long-term outcomes [3].

There is evidence suggesting that TVT-R has excellent treatment rates in cases of SUI caused by urethral hypermobility, intrinsic sphincter deficiency, and recurrent incontinence after retropubic urethropexy. Likewise, TVT-O/TOT has also revealed itself to be effective in the presence of urethral hypermobility and recurrent incontinence after retropubic urethropexy [4-6].

Furthermore, long-term treatment success rates vary from 51 to 88% with a retropubic approach and from 43 to 92% with a transobturator procedure, which may be dependent of the surgical technique and surgeon skills, the sling type and the adequate tension [7]. Nevertheless, about 31.5% of treated patients experience surgical failure with recurrent SUI, of which 17.0% will require repeated sling surgery [8]. Recurrent SUI after any prior sling procedure is defined as urine leakage more than six weeks after the initial success of the first MUS [4,9]. There are multifactorial etiologies that can justify recurrence, including poor surgical technique, inappropriate sling position, and inadequate sling tension when treating intrinsic sphincter deficiency [10].

Treatment options for recurrent SUI after MUS procedures include repeating the midurethral sling, retropubic suspension, urethral bulking agents, pubovaginal sling procedures, shortening of the pre-implanted tape or artificial urethral sphincter. Since MUS is a simple procedure with a high primary success rate, repetition of this technique is an attractive option for initial sling failure [10,11].

In this context, the MUS procedure has been reported to be successful in the treatment of recurrent/persistent SUI regardless of the type of surgical technique previously conducted [4]. According to the literature [12], when a prior midurethral sling fails, a retropubic approach is associated with a higher success rate compared to a transobturator technique for the repeated sling. However, contradictory studies showed that short-term outcomes of TVT-O/TOT are comparable to TVT-R procedures considering the SUI treatment, being associated with fewer intraoperative complications [12]. Therefore, this study aims to compare the outcomes of TVT-R versus TVT-O sling procedures in patients with recurrent SUI who were submitted to a previous incontinence surgery.

## Materials and methods

Study design

This was a comparative retrospective observational study conducted at the Gynecology Department of Unidade Local de Saúde (ULS) of Coimbra, Portugal. We included all patients referred to our department and submitted to a repeated MUS procedure due to recurrent/persistent SUI between January 1st 2019 and December 31st 2023. Both MUS techniques (TVT-R and TVT-O) were considered and compared. Only inside-out approaches were performed in recurrent/persistent SUI in our department during the period of study. Patient medical records were reviewed after obtaining informed consent. Data were collected regarding age, postmenopausal status, parity, body mass index, clinical and surgical characteristics, intra- and post-operative complications, and respective outcomes for a minimum follow-up of 8 months. We also collected information related to previous procedures for SUI and if they were combined with pelvic reconstructive surgery. Patients were included into two groups based on the surgical technique adopted: TVT-R vs TVT-O. The cure was considered in those women who had no further incontinence surgery and had no leaking urine during physical activity, coughing, sneezing, and in the stress test. All investigations included a guarantee of anonymity and were conducted according to the principles expressed in the Declaration of Helsinki.

Statistical analysis

Descriptive and inferential analysis was performed using SPSS Statistics software, version 26.0 (IBM Corp., Armonk, NY). Continuous variables were expressed as median and interquartile ranges (IQR) given their non-normal distribution, and categorical variables were presented as absolute numbers and percentages. Mann-Whitney test and Chi-squared test were used for group comparisons in continuous and categorical variables, respectively. A two-sided p-value<0.05 was considered statistically significant.

## Results

Overall, during the period of study, 860 women were submitted to a midurethral sling procedure, of which 806 (93.7%) placed a transobturator sling and 54 (6.3%) a retropubic sling. Of these, 42 (4.9%) consisted of repeated surgery due to recurrence or persistence of SUI after a previous incontinence surgery (TVT-R in 28 patients (66.7%) and TVT-O in 14 patients (33.3%)).

Sample characterization 

The epidemiological, demographic and clinical patterns of patients who underwent TVT-R or TVT-O sling procedures after a previous incontinence surgery are described in Table 1.

**Table 1:** Epidemiological, demographic and clinical patterns of patients Epidemiological, demographic and clinical patterns of patients submitted to TVT-R vs TVT-O sling procedures after a previous incontinence surgery (N=42). TVT-R: Transvaginal retropubic; TVT-O: tension-free vaginal tape obturator; IQR: Interquartile range; TOT: Transobturator tape * Statistically significant differences for a significance level of 0.05.

	TVT-R	TVT-O	p
Number of patients, n	28	14	
Age, median (IQR), years	54.5 [49.0;62.8]	53.0 [49.5;62.5]	0.968
Postmenopausal status, n (%)	14 (50.0)	9 (64.3)	0.378
Body Mass Index, median (IQR) Kg/m^2^	28.2 [24.9;32.9]	26.0 [23.1; 30.8]	0.292
Comorbidities, n (%)			
Metabolic and Endocrine	5 (17.9)	2(14.3)	0.770
Cardiovascular	14(50.0)	4(28.6)	0.186
Psychiatric	13(46.4)	5(35.7)	0.508
Autoimmune and Vascular	2(7.1)	3 (21.4)	0.178
Neurological	2(7.1)	0 (0.0)	0.306
Multiparity, n (%)	26 (92.9)	14 (100.0)	0.196
Mobile urethra, n (%)	3 (10.7)	12 (85.7)	<0.001*
Primary incontinence surgery, n (%)			
Burch Colposuspension	1(3.6)	0(0.0)	0.490
TOT procedure (outside-in technique)	8(28.6)	0(0.0)	0.032*
TVT-O procedure (inside-out technique)	15(53.6)	8(61.5)	0.632
TVT-R procedure	3(10.7)	1(7.7)	0.762
Minisling	0(0.0)	2(15.4)	0.033*
Two previous SUI surgeries	1(3.6)	2(14.3)	0.204
-Burch colposuspension followed by TVT-O			
-TVT-O followed by TVT-R			
Previous pelvic prolapse surgery, n (%)	1 (3.6)	2 (14.3)	0.204

There were no statistically significant differences among patients undergoing TVT-R vs. TVT-O considering the median age, body mass index, parity, and postmenopausal status (p=non significant (n.s.)). Patient comorbidities were categorized into five main groups: “Metabolic and Endocrine” (thyroid disorders and diabetes); “Cardiovascular” (arterial hypertension and ischemic heart disease/stroke); “Psychiatric” (major depression); “Autoimmune and Vascular” (venous and pulmonary thromboembolism, systemic lupus erythematosus, Behcet's disease, CREST and Raynaud syndrome); and “Neurological” (migraine, Parkinson´s disease and epilepsy). No statistically significant differences were found considering past medical history in patients submitted to TVT-R vs TVT-O (p=n.s.).

Most patients (89.3%) submitted to TVT-R had a fixed urethra, while most patients (85.7%) in the TVT-O group had mobile urethra, presenting significant differences in urethral mobility prior to surgery in both groups (p<0.001). The type of primary incontinence surgery did not differ between groups (p=n.s.), with the exception of TOT procedures that were only performed in patients submitted to TVT-R and mini slings that were only registered in the TVT-O group (p<0.05). Previous concomitant pelvic prolapse surgeries, including colporrhaphies and vaginal hysterectomies, did not also differ between groups (p=n.s.).

Outcomes and complications

The preoperative evaluation, clinical outcomes and postoperative complications of patients submitted to TVT-R vs TVT-O sling after a previous incontinence surgery are summarized in Table 2.

**Table 2:** Preoperative evaluation, clinical outcomes and postoperative complications of patients Preoperative evaluation, clinical outcomes and postoperative complications of patients submitted to TVT-R vs TVT-O sling after a previous incontinence surgery (N=42). TVT-R: Transvaginal retropubic; TVT-O: tension-free vaginal tape obturator; SUI: Stress urinary incontinence.

	TVT-R	TVT-O	p
Urodynamic evaluation prior to surgery, n (%)	17 (60.7)	12 (85.7)	0.085
Sphincter insufficiency in urodynamics, n (%)	2 (7.1)	1 (7.1)	0.763
Revision and/or excision of the previous sling, n (%)	10 (35.7)	7 (50.0)	0.376
Intraoperative and immediate postoperative complications, n (%)	3 (10.7)	1 (7.1)	0.704
Long-term complications, n (%)	6 (21.4)	2 (14.3)	0.571
Complete resolution of SUI after surgery, n (%)	21 (75.0)	12 (85.7)	0.413
Worsening/appearance of urge incontinence, n (%)	5 (17.9)	3 (21.4)	0.783

In most cases, the urodynamic evaluation was performed before surgery in both groups (60.7% vs. 85.7%, p=n.s.), with a similar rate of sphincter insufficiency diagnosis (7.1 % vs 7.1%, p=n.s.). There was a need for revision and/or excision of the previous sling in less than half of the cases in both TVT-R and TVT-O groups (35.7% vs. 50.0%, p=n.s.). The rate of intraoperative and immediate postoperative complications did not differ between groups (10.7% vs. 7.1%, p=n.s.), highlighting the occurrence of three cases of bladder laceration in the group of patients undergoing TVT-R and one case of vaginal laceration in the TVT-O group.

There was a complete resolution of SUI complaints after surgery in 75.0% and 85.7% of cases in TVR-R and TVT-O groups, respectively (p=n.s.). The long-term complications were also similar in both groups (21.4% vs 14.3%, p=n.s.). In the TVT-R group there were four cases of voiding dysfunction and two cases of mesh exposition, one of them requiring mesh surgical revision. On the other hand, in the TVT-O group there were two cases of voiding dysfunction. Lastly, new or worsening complaints of urge incontinence were reported in 17.9% and 21.4% of cases of TVT-R and TVT-O, respectively (p=n.s.).

## Discussion

To our knowledge, this is the first study in Portugal comparing the clinical patterns and surgical outcomes of patients submitted to TVT-R vs TVT-O sling procedures in the treatment of SUI recurrence/persistence after a previous urinary incontinence surgery. In our study, the prevalence of recurrent/persistent SUI surgeries was 4.9%. Other published studies also corroborate these data, reporting similar prevalences (4.3-4.5%) [13,14].

Transobturator and retropubic approaches did not differ in our study in terms of efficacy either in the objective or subjective assessment of SUI. Sun et al. found similar results, although repeated surgeries due to the recurrence or persistence of symptoms were not considered [15]. Miller et al. showed that the failure rate of intrinsic sphincter insufficiency (ISI) treatment using a transobturator technique was noticeably higher when compared to the retropubic approach [16]. In our study, these approaches appeared to be equally effective in the treatment of ISI, although a statistically significant difference was not achieved.

According to some of the oldest reports, repeated MUS is linked to positive clinical outcomes, but it is associated with a marginally increased risk of complications. The exact mechanism of cure after repeated treatment has not been explained, although it is probably the same as it was after the first procedure: urethral closure and bladder neck support under stress [17-19].

Our results revealed that the immediate complications were slightly more common in the retropubic group, though no statistical differences were reported between both techniques. Bladder perforation was the most common complication (10.7%) and occurred only in retropubic surgery, having always been detected and managed intraoperatively, without interference in the surgical procedure, or associated with unfavorable outcomes/major sequelae. Conversely, in a Cochrane review published in 2017, a lower bladder perforation rate of 4.5% was reported [20].

Furthermore, long-term complications were also more common in the retropubic group despite the fact that no statistically significant differences were observed between groups. Voiding dysfunction was the main complication in both groups, particularly in the TVT-R, and mesh exposure occurred only in the retropubic group. This is in accordance with Sun et al. [15] study that described that the transobturator approach, particularly the inside-out technique, was associated with a lower risk of long-term voiding dysfunction when compared with the retropubic approach, mainly due to the natural hammock shape of the transobturator sling. The authors also referred that due to this shape, the transobturator procedure may be associated with a low risk of de novo or urge incontinence [15]. In our study, no statistically significant differences were found between the surgical approaches considering this outcome. In the study of Segal et al. [21], about 9.0% of patients experience de novo urge incontinence symptoms after a midurethral sling, which represents a lower percentage compared to our results (17.9% vs. 21.4%). This may be justified by the fact that, in our study, all patients were submitted to a previous incontinence procedure, probably increasing the occurrence of complications.

Nonetheless, the conclusions from this study should be evaluated within the context of its potential limitations. First, its retrospective design may introduce inherent bias considering some important data that were not considered in this study, namely the period between the primary incontinence surgery and the repeated MUS procedure to treat SUI recurrence.

Second, the importance of pelvic floor ultrasound in evaluating MUS misplacement has been demonstrated, which was not routinely evaluated in our study. Published studies have reported that urodynamics and pelvic floor ultrasound should be performed as an evaluation tool for a complete functional and anatomic investigation before deciding on the next management strategy in patients with SUI persistence or recurrence [22]. These are also feasible exams for predicting outcomes and complications [23]. Unfortunately, in our study, there were few cases of patients with recurrent/persistent SUI that were submitted to a pelvic floor ultrasound, and not all patients performed an urodynamic evaluation before a repeated MUS procedure.

In addition, the short follow-up time considered in our study, which varied between eight months and five years, constitutes another important limitation. This is of particular importance once the follow-up time is needed to assess the lifetime risks for reoperation after MUS surgery, considering the recurrence and persistence of SUI and identifying potential MUS-related complications.

Finally, other important limitations of our study include its small sample size and the derivation of data from a single institution, which makes it pertinent to carry out a multicenter study, including a larger and more diversified sample, with a prospective analysis, to appropriately investigate the outcomes of repeated midurethral slings.

## Conclusions

According to our experience, in the present study, the retropubic midurethral sling was the most performed procedure in the treatment of recurrent/persistent SUI after a previous incontinence surgery. Efficacy rates were high in both surgical techniques, with no statistically significant differences between them. However, although it was expected that TVT-R would result in a higher rate of complications given the greater complexity associated with the surgical technique, no differences were observed between groups in terms of intra- and postoperative complications. Accordingly, the importance of both surgical approaches in the treatment of recurrent/persistent SUI is highlighted, always respecting the patient’s clinical assessment and the type of incontinence procedure previously performed. Also, the high experience of the surgeon plays a crucial role in improving the subjective cure rate and can reduce postoperative complications.

As these conclusions are associated with important clinical and healthcare implications, further studies, particularly with a prospective analysis and a larger study population, should be drawn to best address the efficacy rates and the real medium and long-term outcomes of patients submitted to repeated MUS procedures. In conclusion, we should select the surgical option that provides the best conditions for the treatment of recurrent/persistent SUI, avoiding major complications and always in accordance with the clinical assessment and the patient’s preference.

## References

[1] Haylen BT, de Ridder D, Freeman RM (2010). An International Urogynecological Association (IUGA)/International Continence Society (ICS) joint report on the terminology for female pelvic floor dysfunction. Int Urogynecol J.

[2] Pancholy AB (2020). The transvaginal tape surgery vs. trans-obturator tape for stress urinary incontinence. Gynecology and Pelvic Medicine.

[3] Ulmsten U, Henriksson L, Johnson P, Varhos G (1996). An ambulatory surgical procedure under local anesthesia for treatment of female urinary incontinence. Int Urogynecol J Pelvic Floor Dysfunct.

[4] Su CF, Tsui KP, Tsai HJ, Chen GD (2012). Repeat midurethral sling treatment for prior midurethral sling failure. Gynecol Surg.

[5] Palma PC, Riccetto CL, Dambros M, Herrmann V, Thiel M, Netto NR Jr (2002). Tension-free vaginal tape (TVT): minimally invasive technique for stress urinary incontinence (SUI). Int Braz J Urol.

[6] Rardin CR, Kohli N, Rosenblatt PL, Miklos JR, Moore R, Strohsnitter WC (2002). Tension-free vaginal tape: outcomes among women with primary versus recurrent stress urinary incontinence. Obstet Gynecol.

[7] Ford AA, Taylor V, Ogah J, Veit-Rubin N, Khullar V, Digesu GA (2019). Midurethral slings for treatment of stress urinary incontinence review. Neurourol Urodyn.

[8] Haliloglu B, Karateke A, Coksuer H, Peker H, Cam C (2010). The role of urethral hypermobility and intrinsic sphincteric deficiency on the outcome of transobturator tape procedure: a prospective study with 2-year follow-up. Int Urogynecol J.

[9] Lee KS, Doo CK, Han DH, Jung BJ, Han JY, Choo MS (2007). Outcomes following repeat mid urethral synthetic sling after failure of the initial sling procedure: rediscovery of the tension-free vaginal tape procedure. J Urol.

[10] Huang Y, Chen Z, Shen B (2021). Management and follow-up practices of women with recurrent stress urinary incontinence following transobturator mid-urethral synthetic sling procedure: a 6-year retrospective monocentric university-based study. Front Surg.

[11] Scarpero HM, Dmochowski RR (2004). Sling failures: what's next?. Curr Urol Rep.

[12] Stav K, Dwyer PL, Rosamilia A (2010). Repeat synthetic mid urethral sling procedure for women with recurrent stress urinary incontinence. J Urol.

[13] Morling JR, McAllister DA, Agur W (2017). Adverse events after first, single, mesh and non-mesh surgical procedures for stress urinary incontinence and pelvic organ prolapse in Scotland, 1997-2016: a population-based cohort study. Lancet.

[14] Keltie K, Elneil S, Monga A, Patrick H, Powell J, Campbell B, Sims AJ (2017). Complications following vaginal mesh procedures for stress urinary incontinence: an 8 year study of 92,246 women. Sci Rep.

[15] Sun X, Yang Q, Sun F, Shi Q (2015). Comparison between the retropubic and transobturator approaches in the treatment of female stress urinary incontinence: a systematic review and meta-analysis of effectiveness and complications. Int Braz J Urol.

[16] Miller JJ, Botros SM, Akl MN, Aschkenazi SO, Beaumont JL, Goldberg RP, Sand PK (2006). Is transobturator tape as effective as tension-free vaginal tape in patients with borderline maximum urethral closure pressure?. Am J Obstet Gynecol.

[17] Amaye-Obu FA, Drutz HP (1999). Surgical management of recurrent stress urinary incontinence: a 12-year experience. Am J Obstet Gynecol.

[18] Kane L, Chung T, Lawrie H, Iskaros J (1999). The pubofascial anchor sling procedure for recurrent genuine urinary stress incontinence. BJU Int.

[19] Riachi L, Kohli N, Miklos J (2002). Repeat tension-free transvaginal tape (TVT) sling for the treatment of recurrent stress urinary incontinence. Int Urogynecol J Pelvic Floor Dysfunct.

[20] Ford AA, Rogerson L, Cody JD, Ogah J (2015). Mid-urethral sling operations for stress urinary incontinence in women. Cochrane Database Syst Rev.

[21] Segal JL, Vassallo B, Kleeman S, Silva WA, Karram MM (2004). Prevalence of persistent and de novo overactive bladder symptoms after the tension-free vaginal tape. Obstet Gynecol.

[22] Escura S, Ros C, Anglès-Acedo S, Bataller E, Sánchez E, Carmona F, Espuña-Pons M (2022). Midterm postoperative results of mid-urethral slings. Role of ultrasound in explaining surgical failures. Neurourol Urodyn.

[23] Ghanbari Z, Ayati E, Bastani P, Pourali L, Amini E (2022). Ultrasound evaluation of mid-urethral sling position: a potential predictor of outcomes and adverse effects. Gynecol Obstet Invest.

